# Elevated C-Reactive Protein in Older Men With Chronic Pain: Association With Plasma Amyloid Levels and Hippocampal Volume

**DOI:** 10.1093/gerona/glae206

**Published:** 2024-08-22

**Authors:** Tyler R Bell, Carol E Franz, Kelsey R Thomas, McKenna E Williams, Lisa T Eyler, Imanuel Lerman, Christine Fennema-Notestine, Olivia K Puckett, Stephen M Dorros, Matthew S Panizzon, Rahul C Pearce, Donald J Hagler, Michael J Lyons, Jeremy A Elman, William S Kremen

**Affiliations:** Department of Psychiatry, University of California, San Diego, La Jolla, California, USA; Center for Behavior Genetics of Aging, University of California, San Diego, La Jolla, California, USA; Department of Psychiatry, University of California, San Diego, La Jolla, California, USA; Center for Behavior Genetics of Aging, University of California, San Diego, La Jolla, California, USA; Department of Psychiatry, University of California, San Diego, La Jolla, California, USA; Research Service, VA San Diego Healthcare System, San Diego, California, USA; Department of Psychiatry, University of California, San Diego, La Jolla, California, USA; Department of Psychiatry, University of California, San Diego, La Jolla, California, USA; Department of Anesthesiology, University of California, San Diego, La Jolla, California, USA; Department of Psychiatry, University of California, San Diego, La Jolla, California, USA; Department of Radiology, University of California, San Diego, La Jolla, California, USA; Department of Psychiatry, University of California, San Diego, La Jolla, California, USA; Center for Behavior Genetics of Aging, University of California, San Diego, La Jolla, California, USA; Department of Radiology, University of California, San Diego, La Jolla, California, USA; Department of Psychiatry, University of California, San Diego, La Jolla, California, USA; Center for Behavior Genetics of Aging, University of California, San Diego, La Jolla, California, USA; Department of Psychiatry, University of California, San Diego, La Jolla, California, USA; Center for Behavior Genetics of Aging, University of California, San Diego, La Jolla, California, USA; Department of Anesthesiology, University of California, San Diego, La Jolla, California, USA; Department of Radiology, University of California, San Diego, La Jolla, California, USA; Department of Psychology, Boston University, Boston, Massachusetts, USA; Department of Psychiatry, University of California, San Diego, La Jolla, California, USA; Center for Behavior Genetics of Aging, University of California, San Diego, La Jolla, California, USA; Department of Psychiatry, University of California, San Diego, La Jolla, California, USA; Center for Behavior Genetics of Aging, University of California, San Diego, La Jolla, California, USA; (Medical Sciences Section)

**Keywords:** Amyloid-beta, Chronic pain, Hippocampus, Inflammation, Plasma biomarkers

## Abstract

**Background:**

Chronic pain leads to tau accumulation and hippocampal atrophy, which may be moderated through inflammation. In older men, we examined associations of chronic pain with Alzheimer’s disease (AD)-related plasma biomarkers and hippocampal volume as moderated by systemic inflammation.

**Methods:**

Participants were men without dementia. Chronic pain was defined as moderate-to-severe pain in 2+ study waves at average ages 56, 62, and 68. At age 68, we measured plasma amyloid-beta (Aβ42, *n* = 871), Aβ40 (*n* = 887), total tau (t-tau, *n* = 841), and neurofilament light chain (NfL, *n* = 915), and serum high-sensitivity C-reactive protein (hs-CRP, *n* = 968), a marker of systemic inflammation. A subgroup underwent structural MRI to measure hippocampal volume (*n* = 385). Analyses adjusted for medical morbidities, depressive symptoms, and opioid use.

**Results:**

Chronic pain was related to higher Aβ40 (β = 0.25, *p* = .009), but hs-CRP was unrelated to AD-related biomarkers (*p*s > .05). There was a significant interaction such that older men with both chronic pain and higher levels of hs-CRP had higher levels of Aβ42 (β = 0.36, *p* = .001) and Aβ40 (β = 0.29, *p* = .003). Chronic pain and hs-CRP did not interact to predict levels of Aβ42/Aβ40, t-tau, or NfL. Furthermore, there were significant interactions such that Aβ42 and Aβ40 were associated with lower hippocampal volume, particularly when levels of hs-CRP were elevated (hs-CRP × Aβ42: β = −0.19, *p* = .002; hs-CRP × Aβ40: β = −0.21, *p* = .001), regardless of chronic pain status.

**Conclusions:**

Chronic pain was associated with higher plasma Aβ, especially when hs-CRP was also elevated. Higher hs-CRP and Aβ levels were both related to smaller hippocampal volumes. Chronic pain, when accompanied by systemic inflammation, may elevate the risk of neurodegeneration in AD-vulnerable regions.

One in five older adults experiences chronic pain, linked to accelerated cognitive decline and a doubled risk of Alzheimer’s disease and related dementias (ADRD) (1,2). Chronic pain has led to AD-related pathology in a mouse model showing increased tauopathy and hippocampal atrophy (3), and a Mendelian randomization study showed a causal link of chronic pain with AD risk (4). Higher levels of neuroinflammation and tauopathy have also been observed in older adults with chronic pain compared to those without chronic pain (5). The connection between chronic pain and the risk of ADRD calls for investigation of the association between chronic pain and AD-related biomarkers.

Amyloid-beta Aβ42 and tau serve as integral biomarkers for early AD detection (6). Aβ42 is typically reduced in cerebrospinal fluid and blood as amyloid plaques accumulate in the brain (7). This trend is examined via the Aβ42/Aβ40 ratio to account for non-AD-specific amyloid proteins (8). Multiple forms of tau proteins also accumulate inside and outside the brain during the preclinical AD stage (9). Biomarkers can also capture evidence of atrophy due to AD pathology, such as neurofilament light (NfL), a protein released during neurodegeneration (10). Due to elevated AD risk, it is expected that chronic pain correlates with lower Aβ42 and Aβ42/Aβ40 and higher tau in plasma. Chronic pain is also hypothesized to correlate with higher NfL due to neurodegeneration.

Chronic pain leads to long-term systemic inflammation, involving elevated cytokines and C-reactive protein (CRP) (11). Elevated CRP, positively correlated with pain severity and disability, is observed in individuals with chronic pain (12). Systemic inflammation, associated with AD-indicative levels of plasma biomarkers, may be a mechanism by which chronic pain causes higher AD pathology (13). Systemic inflammation may also influence changes in plasma biomarkers related to AD independent of cerebral amyloid deposition. Specifically, plasma Aβ42 and Aβ40 levels often rise with age and the presence of comorbidities (14). This increase is largely due to plasma Aβ42 and Aβ40 being released from activated platelets as an inflammatory response, effectively functioning as an additional cytokine (15). In this scenario, higher CRP may be related to greater plasma Aβ42 and Aβ40 levels in individuals with chronic pain due to systemic inflammation. Still, this could indicate a unique form of inflammation contributing to neurodegeneration in AD-vulnerable brain regions in older adults with chronic pain, perhaps due to indirect processes such as crosstalk of peripheral inflammation across the blood-brain barrier (BBB) to induce neuroinflammation (16).

Here, we examined how chronic pain and hs-CRP jointly relate to AD-related biomarkers. If chronic pain and systemic inflammation lead to AD pathology, than we predict that individuals with both chronic pain and elevated hs-CRP would show lower plasma Aβ42 and Aβ42/Aβ40 and higher total tau (t-tau) and NfL levels, consistent with higher AD risk (17). If chronic pain elevates plasma Aβ production due to systemic inflammation and not necessarily AD pathology, then we can expect that individuals with both chronic pain and elevated hs-CRP would show higher plasma Aβ42 and Aβ40. We also investigated how elevated hs-CRP and AD-related biomarkers predicted hippocampal volume. We expected that heightened levels of hs-CRP and more AD-indicative plasma biomarker levels would correspond to smaller hippocampal volumes, with these associations being strongest in individuals with, compared to those without, chronic pain. Our analyses consider the moderating roles of depressive symptoms, pain inference with functional activities, *APOE*-ε4 status, and presence of mild cognitive impairment (MCI).

## Method

### Participants

Participants were men in the Vietnam Era Twin Study of Aging (VETSA). VETSA is a longitudinal study assessing men at average ages 56, 62, and 68—an approximately 12-year time span. VETSA randomly recruited 1 291 participants from the Vietnam Era Twin Registry (VETR), a nationally distributed registry of nonpatient community-dwelling male–male twin pairs all of whom served in the United States military at some time between 1965 and 1975 (18). Nearly 80% of VETSA participants report no combat exposure. Note also that VETSA is not a Veterans’ Administration patient sample. They are now men living throughout the United States, who are similar in terms of health, education, and lifestyle to American men in their age cohort based on census data (19). Wave 1 eligibility requirements included (a) being between the ages of 51 and 59 at enrollment, and (b) both members of a twin pair agreeing to participate in the baseline assessment. Details of ascertainment and data collection protocols are described elsewhere (20). Wave 1 data collection occurred between 2003 and 2007 with follow-up data collections at wave 2 (2009–2014) and wave 3 (2016–2019). VETSA follow-up waves included approximately 82% of the original participants as well as age-matched attrition replacement participants randomly recruited from the same VETR cohort. Most data are publicly accessible to researchers by request (https://psychiatry.ucsd.edu/research/programs-centers/vetsa/researchers.html). VETSA procedures were approved by the Institutional Review Boards at the University of California, San Diego (UCSD), and Boston University. Protocols were identical at each site. The outcome measures for the present study were from wave 3 (2016–2019) when average age was 68. Biomarkers for participants at both sites were assessed at wave 3. MRIs at wave 3 were performed at the UCSD site only.

For the first aim, we examined how chronic pain and hs-CRP related to AD-related biomarkers. Chronic pain status was obtained at wave 3 by assessing pain reporting history at the previous 2 waves. Values of hs-CRP and AD-related biomarkers were obtained from blood collection at wave 3. There were 1 196 participants at wave 3 with available health data. We removed 66 individuals with reports of a physician’s diagnosis of stroke, resulting in a sample of 1 130. Stroke was chosen as a major health condition that can alter plasma biomarker and imaging results (21,22). Other major medical comorbidities were statistically adjusted for rather than excluded to preserve sample size. We further removed individuals with only one wave of data (*n* = 196), as 2 time points were required to assess the presence of chronic pain. Of the remaining individuals, data were available for Aβ42 (*n* = 871), Aβ40 (*n* = 887), total tau (t-tau, *n* = 841), NfL (*n* = 915), and hs-CRP (*n* = 968). Participants with chronic pain, hs-CRP, and at least one AD-related biomarker available (*n* = 887) had an average age of 67.56 years (*SD* = 2.53, range = 61.37–73.25).

For the second aim, we examined a subsample of participants who underwent MRIs of the brain (*n* = 434), meeting standard MRI safety inclusion criteria (eg, no metal in the body) and completed imaging of the hippocampus. MRI participants with inadequate hippocampal segmentation data (*n = *47) were removed from the sample, as were individuals who did not have at least 2 time points of data on pain (*n* = 30), resulting in a final sample of 387 individuals for analyses of hippocampal volume. Of this subsample, most participants had data on Aβ42 (*n* = 331), Aβ40 (*n* = 332), t-tau (*n* = 317), NfL (*n* = 333), and hs-CRP (*n* = 348). Participants with data on chronic pain, hs-CRP, at least one AD-related biomarker, and quality data on hippocampal volume (*n* = 333) had an average age of 67.29 years (*SD* = 2.65, range = 61.37–71.00).

### Measures

#### Chronic pain

Pain was assessed using the SF-36 Quality of Life (Version 1.0) Bodily Pain scale which was given at each assessment wave (23). We used the pain severity item, “How much pain severity have you had during the past 4 weeks.” Severity was rated on a 6-point Likert-type scale: “None” (1),“Very Mild” (2),“Mild” (3),“Moderate” (4),“Severe” (5),“Very Severe” (6). Chronic pain was defined as moderate-to-very severe pain (values 4–6 on the scale) at wave 3 and at least 1 of the 2 previous waves. Pain reported over 2 or more waves (approximately 6–12 years) fits common definitions of chronic pain as moderate-to-severe pain severity that lasts or recurs 3 or more months (24).

#### Pain interference

In addition, we assessed functional difficulties due to pain, known as pain interference (23). We specifically used an item on the SF-36 version 1 that asks, “During the past 4 weeks, how much did pain interfere with your normal work (including both work outside the home and housework)? Interference is rated on a 6-point Likert-type scale: Severity was rated on a 6-point Likert-type scale: “Not at all” (1),“Slightly” (2),“Moderately” (3),“Quite a Bit” (4),“Extremely” (5).

#### C-reactive protein

High-sensitivity serum C-reactive protein (hs-CRP) levels were assessed with nephelometry. Assays were conducted by Quest Diagnostics Inc./Nichols Institute, San Juan Capistrano, CA, USA. For analytical purposes, we used a square root transformed measure of the continuous measure to account for a positive skew. This was then *z*-scored for easier interpretability and winsorized at 3 standard deviations to avoid undue influence of extreme outliers. Furthermore, for illustration purposes, we further categorized hs-CRP values into levels based on tertiles including low (0 to 0.40 mg/dL), mild (0.40 to 2.60 mg/dL), moderate (2.61 to 4.70 mg/dL), and high levels (4.71 to 19.00 mg/dL). Values higher than 19 were winsorized as this is 3 *SD* above the mean. Note that moderate and high categories most closely correspond to clinically significant levels (>3 mg/dL) (25).

#### AD-related biomarkers

AD-related biomarkers included plasma levels of Aβ40, Aβ42, t-tau, and NfL. Blood was collected after fasting beginning by 9:00 pm the night before the study appointment. Specimens were collected between 8:00 am and 8:30 am the next morning. Plasma levels of t-tau, Aβ40, and Aβ42 were assayed using the high-throughput Simoa Human Neurology 3-plex A (N3PA) on the Simoa HD-X platform (Quanterix, Billerica, MA, USA). Sample handling and assays were performed following clinical trial standard operating procedures and manufacturer instructions. This approach has been developed by Winston et al. and used in multiple publications from the laboratory of Dr. Robert Rissman (26–29). Plasma NfL was assayed using the Simoa NfL Advantage Kit on the Simoa HD-X platform (Quanterix) provided by the Alzheimer’s Therapeutic Research Institute Biomarker Core at the University of Southern California (PI: Dr. Robert Rissman) (26). Values were excluded if there was presence of hemolysis and/or a coefficient of variance for plasma concentrations >0.20. Each of these 4 biomarker measures was preadjusted for site and storage time. Values of each were log-transformed to adjust for positive skew, *z*-scored for easier interpretability, and winsorized at 3 standard deviations to avoid the influence of extreme outliers (results were not different when using non-winsorized scores). Log-transformation was chosen to correct for positive skew. An Aβ42/Aβ40 ratio was calculated after transformation as a measure thought to be more specific to AD pathology compared to either Aβ42 or Aβ40 level alone (lower level is typically thought to indicate greater cerebral amyloid deposition) (30).

##### MRI acquisition and processing of hippocampal volume

We previously described our MR imaging procedures (31). In brief, participants completed scans in GE 3T Discovery 750x scanners (GE Healthcare, Waukesha, WI, USA) with an 8-channel phased-array head coil. We acquired sagittal T_1_-weighted 3D fast spoiled gradient echo sequences (TE = 3.164 ms, TR = 8.084 ms, TI = 600 ms, flip angle = 8°, matrix = 256 × 192, in-plane resolution = 1 × 1 mm, slice thickness = 1.2 mm, slices = 172). MRIs were processed using methods previously described but utilizing the latest software (32). In brief, processing involved atlas-based volumetric segmentation to derive hippocampal volume using FreeSurfer version 6.0 (http://surfer.nmr.mgh.harvard.edu). Quality of subcortical segmentations was checked using visual review, and participants with inaccurate segmentations (defined by obvious overestimation or underestimation on the segmentation atlas via a detailed lab protocol) were removed from analysis (*n* = 47). Hippocampal volume was preadjusted (residualized) for an individual’s estimated intracranial volume.

##### Classification of mild cognitive impairment

Mild cognitive impairment was diagnosed using the Jak-Bondi approach with the 18 neuropsychological tests covering 6 cognitive domains (33–37). The impairment criterion was scoring >1.5 *SD*s below normative means on 2 or more tasks within a cognitive domain. Data were also collected on general cognitive ability, measured with the Armed Forces Qualifying Test (AFQT) at average age 20 (38–40). Prior to calculating these cutoffs, scores were adjusted for early adulthood general cognitive ability (measured at average age 20) to ensure that they reflected a decline in performance rather than just longstanding low ability. MCI included the presence of amnestic and nonamnestic MCI.

##### Covariates

We additionally measured covariates that could confound the associations with persistent pain and other measures of interest, including age, race/ethnicity (non-Hispanic White vs other), lifetime education (years), depressive symptoms indexed using the 20-item Center of Epidemiological Studies-Depression scale (CES-D) (41), medical morbidities (total number of reported 15 major health conditions from a modified Charlson Index) (42), and use of opioid medication based on reports of medications taken in the medical history interview (yes/no). For sensitivity analyses, *APOE*-ε4 allele status was also used as a covariate and as a moderator. *APOE* genotype was determined from blood samples using established methods (43). All genotypes were independently determined twice by laboratory personnel at the VA Puget Sound Healthcare System who were blind to the genotype and the identity of the co-twin. For our analyses, we coded *APOE* by the presence or absence of at least one ε4 allele. As mentioned, hippocampal volume was preadjusted for intracranial volume; thus, intracranial volume was not included as a covariate.

### Statistical Analysis

We compared key covariates between older men with and without chronic pain for descriptive purposes. We used the package *lmer* in *R 2.4.3* to conduct linear mixed models. Linear mixed models were used to test main hypotheses by examining (1) interactive associations of chronic pain and hs-CRP with AD-related biomarkers and (2) interactive associations of hs-CRP and AD-related biomarkers with hippocampal volume among older men with and without chronic pain. Models included a random intercept for twin pair to account for family relatedness. The equation for the first objective to examine how chronic pain and hs-CRP interact predict AD-related biomarkers was as follows: *Y*_*ij*_(outcome)~ β0_*ij*_ + β1(chronic pain status)_*ij*_ + β2(hs-CRP)_*ij*_ + β3(age)_*ij*_ + β4(race)_*ij*_ + β5(lifetime education)_*ij*_ + β6(depressive symptoms)_*ij*_ + β7(medical morbidities)_*ij*_ + β8(number of opioid medications reported)_*ij*_ + β8(chronic pain status × hs-CRP)_*ij*_ + ε_*ij*_; *i* = individual participants, *j* = twin pairs. The equation for the second objective to assess how the interaction of hs-CRP and AD-related biomarkers predict hippocampal volume was as follows: *Y*_*ij*_(hippocampal volume)~ β0_*ij*_ + β1(hs-CRP)_*ij*_ + β1([AD-related biomarker])_*ij*_ + β2(age)_*ij*_ + β3(race)_*ij*_ + β4(lifetime education)_*ij*_ + β5(depressive symptoms)_*ij*_ + β6(medical morbidities)_*ij*_ + β7(number of opioid medications reported)_*ij*_ + β8(hs-CRP) × [AD-related biomarker])_*ij*_ + ε_*ij*_; *i* = individual participants, *j* = twin pairs. A 3-way interaction term tested whether or not interactions between hs-CRP and AD-related biomarkers were moderated by chronic pain status (eg, hs-CRP × AD-related biomarker × chronic pain status)_*ij*_. Pattern of results did not differ when adjusting for *APOE*-ε4 status, MCI status, depressive symptoms, and pain interference as covariates. For all equations, we additionally conducted sensitivity analyses to examine the moderating effect of depressive symptoms, pain interference, *APOE*-ε4 status, and MCI status (see Supplementary Tables 9 and 10). Statistical significance was set to an α of 0.05, and 95% confidence intervals were calculated.

## Results

### Descriptive Statistics

Descriptive statistics are shown in Table 1. Overall, 20.6% of older men reported chronic pain (*n* = 192). Older men with chronic pain tended to be older (*t*(932) = 2.75, *p* = .006, *d* = 0.18) and have fewer years of education (*t*(1 050) = 4.33, *p* < .001, *d* = 0.27) than older men without chronic pain. Older men with chronic pain also reported more depressive symptoms (*t*(1 047) = 7.57, *p* < .001, *d* = 0.45), medical morbidities (χ^2^(1) = 48.48, *p* < .001, *d* = 0.56), and a greater number used an opioid (χ^2^(1) = 78.80, *p* < .001, *d* = 0.43) than older men without chronic pain. Men with chronic pain showed higher levels of Aβ40 (*t*(885) = 16.88, *p* < .001, *d* = 1.04) and hs-CRP (*t*(966) = −2.74, *p* = .006, *d* = 0.09) compared to participants without chronic pain. Looking at MCI, 38 (20%) men with chronic pain had MCI (amnestic MCI: *n* = 23; nonamnestic MCI: *n* = 15). This was slightly higher than for men without chronic pain (*n* = 139, 16%; amnestic MCI: *n *= 105; nonamnestic MCI: *n* = 34), although overall nonsignificant (χ^2^(1) = 1.48; *p* = .224; amnestic MCI: χ^2^(1) = 1.12, *p* = .290; nonamnestic MCI: χ^2^(1) = 5.26, *p* = .022).

**Table 1.** Descriptive Statistics and Bi-Variate Correlations With hs-CRP by Chronic Pain Status

		Overall	No Chronic Pain	Chronic Pain		
		(*n*_max_ = 1 052)	(*n*_max_ = 860)	(*n*_max_ = 192)	*t*/χ^2^	*p* Value
Age (years)	*M* (*SD*)	67.6 (2.53)	67.6 (2.55)	67.2 (2.43)	2.04	.041
	*Total n*	1 052	860	192		
Lifetime education (years)	*M* (*SD*)	14.0 (2.13)	14.1 (2.14)	13.4 (2.00)	4.33	<.001
	*Total n*	1 052	860	192		
Depressive symptoms (CES-D)		7.01 (7.53)	6.03 (6.70)	11.4 (9.31)	−7.57	<.001
		1 049	857	192		
Physical morbidities						
0	*f*(*n*)	177 (16.8%)	168 (19.5%)	9 (4.7%)	48.48	<.001
1	*f*(*n*)	293 (27.9%)	258 (30.0%)	35 (18.2%)		
2+	*f*(*n*)	582 (55.3%)	434 (50.5%)	148 (77.1%)		
	*Total n*	1 052	860	192		
Opioid use						
No	*f*(*n*)	981	827	154	78.80	<.001
Yes	*f*(*n*)	58	22	36		
	*Total n*	1 039	849	190		
hs-CRP	*M* (*SD*)	−0.05 (0.93)	−0.09 (0.90)	0.15 (1.04)	−2.74	.006
	*Total n*	968	796	172		
Aβ42/Aβ40 ratio	*M* (*SD*)	0.10 (0.46)	0.10 (0.47)	0.08 (0.38)	0.55	.582
	*Total n*	871	724	147		
Aβ42	*M* (*SD*)	−0.01 (0.92)	−0.03 (0.89)	0.10 (1.05)	−1.44	.151
	*Total n*	871	724	147		
Aβ40	*M* (*SD*)	−0.03 (0.92)	−0.06 (0.89)	0.15 (1.04)	−16.88	<.001
	*Total n*	887	738	149		
t-tau	*M* (*SD*)	−0.07 (0.83)	−0.08 (0.79)	0.0230 (0.998)	−1.14	.255
	*Total n*	841	698	143		
NfL	*M* (*SD*)	−0.04 (0.76)	−0.06 (0.73)	0.071 (0.90)	−1.70	.089
	*Total n*	915	757	158		
Hippocampal volume	*M* (*SD*)	0.02 (0.99)	0.07 (0.96)	−0.28 (1.16)	2.13	.034
	*Total n*	387	331	56		
*APOE*-ε4					0.14	.709
ε4−	*f*(*n*)	659 (62.6%)	522 (60.7%)	137 (71.4%)		
ε4+	*f*(*n*)	269 (25.6%)	216 (25.1%)	53 (27.6%)		
	*Total n*	928	738	190		

*Notes*: Biomarkers were log-transformed, *z*-scored, and winsorized at 3 standard deviations. Specific *n* sample sizes for hs-CRP, biomarkers, and hippocampal volumes are described in the main text. Bolded variable names are significantly different at *p* < .05 between older adults with no chronic pain and with chronic pain. Aβ42 = amyloid-beta 42; Aβ40 = amyloid-beta 40; CES-D = Center of Epidemiological Studies-Depression scale; hs-CRP = C-reactive protein; *SD* = standard deviation.

### Biomarkers by Chronic Pain Status and hs-CRP

**Figure 1.**
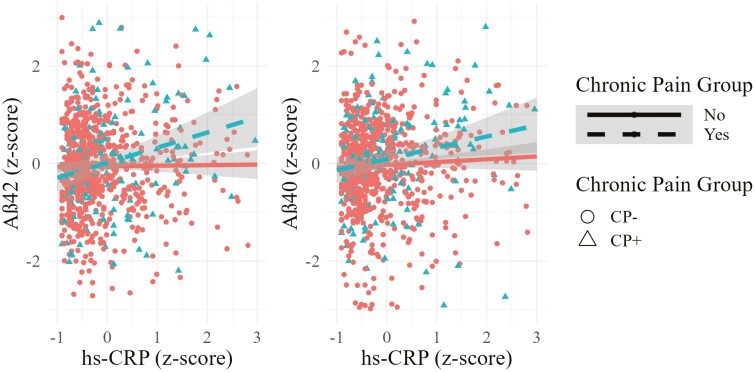
Association of hs-CRP with Aβ42 and with AB40 in men with chronic pain and without chronic pain (Aβ42 *n* = 871; Aβ40 *n* = 887). Aβ42 = amyloid-beta 42; Aβ40 = amyloid-beta 40; hs-CRP = high-sensitivity serum C-reactive protein. hs-CRP and Aβ42 and hippocampal volume values are *z*-scored for ease of interpretability.

**Figure 2.**
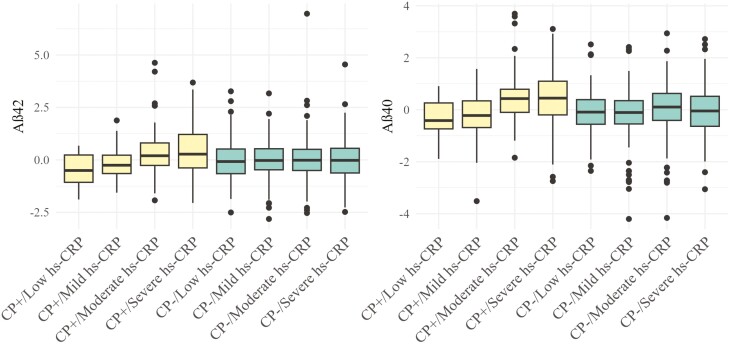
Differences in Aβ42 level and Aβ40 by severity of hs-CRP level in men with chronic pain and without chronic pain (CP+ versus CP−; Aβ42 *n* =871; Aβ40 n = 887). Aβ42 = amyloid-beta 42; Aβ40 = amyloid-beta 40; hs-CRP = high-sensitivity serum C-reactive protein. Aβ42 values were *z*-scored for ease of interpretability. hs-CRP levels represent tertiles of low (0 to 0.40 mg/dL), mild (0.40 to 2.60 mg/dL), moderate (2.61 to 4.70 mg/dL) and high values (4.71 to 19.00* mg/dL, *upper values winsorized at 3 *SD* or 19.00).

Linear mixed models revealed significant interactions between chronic pain status and hs-CRP level when predicting Aβ42 (β = 0.36, 95% CI: 0.15 to 0.56, *p* = .001) and Aβ40 (β = 0.29, 95% CI: 0.10 to 0.49, *p* = .003) such that older men with chronic pain showed higher Aβ42 and Aβ0 levels when hs-CRP levels were higher (see Table 2). These interactions are illustrated continuously in Figures 1 and 2. Interactions of chronic pain and hs-CRP did not significantly predict levels of Aβ42/Aβ40 ratio (*p* = .999), t-tau (*p* = .802), or NfL (*p* = .375), as shown in Supplementary Tables 1–3 and illustrated in Supplementary Figures 1–3. Looking at main effects, hs-CRP was not significantly related to any AD-related biomarker when chronic pain was not present (*p*s > .05). Meanwhile, chronic pain was related to higher Aβ40, even after controlling for hs-CRP status (β = 0.25, 95% CI: 0.06, 0.43, *p* = .009), but not other AD-related biomarkers (*p*s > .05). Depressive symptoms, pain interference, *APOE*-ε4 status, or MCI status were not significant moderators (*p*s > .10; see Supplementary Table 9).

**Table 2.** Linear Mixed Model Predicting Aβ Levels From Interaction of Chronic Pain and hs-CRP Level

	Aβ42			Aβ40		
Predictors	β	CI	*p* Value	β	CI	*p* Value
(Intercept)	−0.15	−0.26, −0.03	.012	−0.13	−0.24, −0.01	.031
Chronic pain	0.16	−0.03, 0.35	.101	0.24	0.06, 0.43	.009
Age	0	−0.08, 0.07	.926	0.03	−0.04, 0.11	.371
hs-CRP	0.02	−0.06, 0.11	.613	0.03	−0.05, 0.12	.469
Physical morbidities	0.07	0.02, 0.12	.008	0.05	−0.00, 0.10	.055
Opioid use	−0.04	−0.34, 0.26	.785	−0.10	−0.39, 0.19	.490
Chronic pain × hs-CRP	0.36	0.15, 0.56	.001	0.29	0.10, 0.49	.003
Random effects						
Level 1 Error (σ^2^)	0.61			0.53		
Level 2 Error (τ00)	0.36			0.43		
ICC	0.37			0.45		
*Total* *n*	871			887		
Marginal *R*^2^/Conditional *R*^2^	0.030/0.388			0.027/0.462		

*Notes*: Aβ42 = amyloid-beta 42; Aβ40 = amyloid-beta 40; hs-CRP = C-reactive protein; ICC = intraclass coefficient, represents the percent of outcome variability explained by people being nested within twin pairs; σ^2^ = Level 1 error, error across participants; τ00 = Level 2 error, error within twin pairs. Model results did not change when adding *APOE*-ε4 status as a covariate or a moderator (nonsignificant moderator).

### Hippocampal Volume

**Figure 3.**
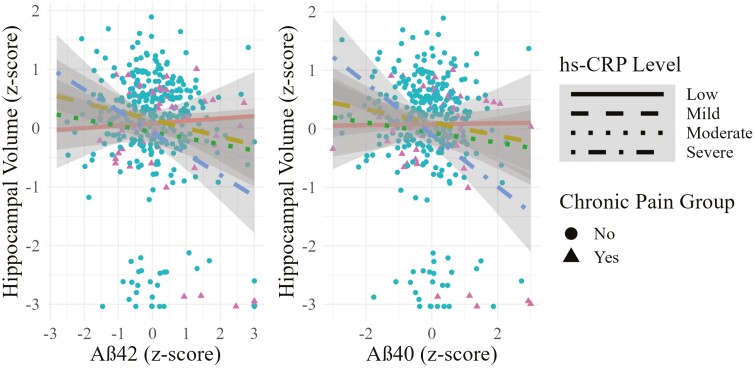
The association of Aβ42 and hippocampal volume by hs-CRP level (*n* = 331). Aβ42 = amyloid-beta 42; Aβ40 = amyloid-beta 40; hs-CRP = high-sensitivity serum C-reactive protein. Aβ42 and hippocampal volume values were *z*-scored for ease of interpretability. hs-CRP levels represent tertiles of low (0 to 0.40 mg/dL), mild (0.40 to 2.60 mg/dL), moderate (2.61 to 4.70 mg/dL) and high values (4.71 to 19.00* mg/dL, *upper values winsorized at 3 *SD* or 19.00). The figure illustrates that the relationship of Aβ42 and hippocampal volume is steeper (ie, stronger) at higher levels of hs-CRP.

We examined how interactions of AD-related biomarkers and hs-CRP related to hippocampal volume among individuals with and without chronic pain. A linear mixed model revealed that older men who had both higher levels of Aβ42 and higher levels of hs-CRP had smaller hippocampal volumes (β = −0.19, 95% CI: −0.31, −0.07, *p* = .002, see Supplementary Table 4 and Supplementary Figure 4). Older men with higher levels of both Aβ40 and hs-CRP also had smaller hippocampal volumes (β = −0.22, 95% CI: −0.35, −0.08, *p* = .002, see Supplementary Table 5 and Supplementary Figure 4). This is illustrated in Figure 3. This was also true when looking at interactions of hs-CRP with levels of t-tau (β = −0.12, 95% CI: −0.24, −0.01, *p* = .048, see Supplementary Table 6 and Supplementary Figure 4). The Aβ42/Aβ40 ratio (*p* = .671, see Supplementary Table 7 and Supplementary Figure 5) and NfL (*p* = .350, see Supplementary Table 8 and Supplementary Figure 6) did not significantly interact with hs-CRP to predict hippocampal volume. A 3-way interaction term added to the model showed that significant interaction effects of hs-CRP with Aβ42, Aβ40, or t-tau did not differ by chronic pain status (*p*s > .10). However, as shown in Supplementary Figures 4 and 5, older men with chronic pain were more likely to be in the higher ranges for values of hs-CRP, Aβ42, and Aβ40. Depressive symptoms, pain interference, *APOE*-ε4 status, or MCI status were not significant moderators (*p*s > .10; see Supplementary Table 10).

## Discussion

Chronic pain has been associated with an increased risk of ADRD, but the exact mechanisms remain unclear (44). One previous study has found an association of chronic pain with neuroinflammation and tauopathy in older adults, but the role of systemic inflammation is lesser known (45). In the present study, we examined the association of chronic pain and systemic inflammation with AD-related biomarkers in early older men (age range: 61.37 to 73.25 years) without dementia as it may provide insights into the pathogenesis of AD among individuals with chronic pain. Overall, we found that chronic pain was related to higher levels of plasma Aβ40; and chronic pain related to Aβ42 and Aβ40 when accompanied by higher levels of hs-CRP. We further found that participants with elevated levels of both hs-CRP and Aβ42 (or Aβ40), in addition to being more likely to have chronic pain, also had the smallest hippocampal volumes. Similar findings were seen when examining associations with chronic pain interference and across *APOE*-ε4 and MCI status. These findings highlight the importance of considering peripheral inflammation when assessing plasma AD-related biomarkers in people with chronic pain. Below we discuss how our study findings fit into previous literature on chronic pain and AD risk and speculate on potential mechanisms to guide future inquiries.

Previous studies of chronic pain and AD risk have involved large-scale analyses of medical records, showing a positive association between chronic pain and incident diagnosis of ADRD (2). If chronic pain indeed increases AD-specific risk, then we should also see associations with preclinical indicators including atrophy in AD-related brain structures and AD-related biomarker levels before dementia diagnosis. Zhao et al. (2023) showed that chronic pain is associated with cognitive decline before dementia diagnosis and this decline was mediated by hippocampal atrophy (46). However, to our knowledge, no prior studies have examined chronic pain and AD-related biomarkers.

Here, we showed that older men with chronic pain and higher levels of systemic inflammation show higher levels of plasma Aβ42. However, higher plasma Aβ42 is counterintuitive to prevailing notions regarding AD risk in which individuals at elevated risk for AD exhibit lower Aβ42 levels in cerebrospinal fluid as amyloid is trapped in the brain (7,8). Our observation of elevated plasma Aβ42 prompts a reevaluation of our understanding that plasma Aβ42 has a normal association with cerebral amyloid in men with chronic pain. It may either follow a nonlinear pattern and be mostly driven by peripheral inflammation altogether.

One mechanism may be that plasma Aβ42 may be elevated in the earliest preclinical stages of AD (47–50). Although lower levels of cerebrospinal fluid or plasma Aβ42 are typically associated with greater pathology, a nonlinear or biphasic pattern has been demonstrated in at least 3 independent samples (51). In those samples, cerebrospinal fluid Aβ42 was positively associated with tau in adults aged 70 and under but negatively associated in those over 70 (51). Given the age of the VETSA sample, the positive correlation between chronic pain and Aβ42 might become negative in later waves of the study. Furthermore, extracerebral Aβ42 may not be the best marker of amyloid deposition in men with systemic inflammation. In a recent study, men with higher values of systemic inflammation, measured by hs-CRP, showed greater CSF Aβ42 (52). Counterintuitive to prevailing notions, these men with high hs-CRP levels had higher amyloid deposition on PET in later life. This suggests that basing interpretation of extracerebral Aβ42 as a marker of cerebral amyloid deposition in men with systemic inflammation on a simple linear function may be inadequate. Our article highlights the influence of systemic inflammation on plasma Aβ42 that may contribute to inconsistent associations of plasma Aβ42 with AD risk found previously (53,54).

Chronic pain and systemic inflammation may worsen overall brain health, particularly by heightening plasma Aβ42 and Aβ40 levels as an additional inflammatory response. Plasma Aβ42 and Aβ40 is elevated as an innate immune response to inflammation (55), which appears to be due to activated blood platelets that contribute significantly to the production of over 90% of plasma Aβ42 and Aβ40 (56). As plasma Aβ accumulates, systemic inflammation delivers a substantial payload of cytokines, chemokines, and lipid mediators to the BBB (57). These inflammatory agents compromise BBB integrity, resulting in widened endothelial spaces, providing a route for proinflammatory molecules to induce neuroinflammation through reactive microglia and astrocytes, which is known to facilitate cerebral amyloid plaque deposition and tau phosphorylation (16). Aβ42 and Aβ40 may act as exacerbating cytokines or act uniquely in helping migrate leukocytes across the BBB (58). Our finding that individuals with heightened levels of both hs-CRP and Aβ42 or Aβ40 also had the smallest hippocampal volume supports the notion that systemic inflammation involving heightened plasma Aβ42 or Aβ40 may play an important role in setting the stage for worse brain health in older men with chronic pain. Systemic inflammation and worsened brain health contributing to neurodegeneration in AD-vulnerable regions like the hippocampus. The role of AD processes will require more direct measures such as PET imaging or post-mortem immunoassaying of brain tissue.

There is a possibility that chronic pain elevates dementia risk due to inflammation-related tauopathy and not necessarily AD-related amyloid deposition. In a study of adults older than in the present study, levels of cerebrospinal fluid glial fibrillary acidic protein—a marker of microglia activity and neuroinflammation in the brain—were higher in older men with chronic pain compared to those without chronic pain, but those with chronic pain were not more likely to show amyloidosis (5). Instead, they were more likely to have tauopathy without the presence of amyloidosis (suspected non-Alzheimer’s pathophysiology or SNAP). Our findings might also be explained by this process, while also highlighting an important role of systemic inflammation. Higher levels of systemic inflammation have been shown to activate microglia that produce proinflammatory cytokines and exacerbate tau pathology (59). This is thought to be facilitated by BBB leakage that allows the entry of inflammatory cells or molecules into the brain (60). Systemic inflammation can also lead to the recruitment of plasma immune cells, such as T cells, into the brain, contributing to neuroinflammation and tau pathology (61). As an important limitation, this other study classified amyloidosis using CSF markers. As mentioned, this may not be suitable for certain groups, particularly men with systemic inflammation (52).

Our findings should be taken in the context of specific study limitations and strengths. We cannot directly address causality due to the cross-sectional observational nature of our study. However, an animal model showed that chronic pain did lead to AD pathology and hippocampal neurodegeneration (3). Furthermore, without longitudinal data collection we cannot understand the dynamic temporal associations of chronic pain, hs-CRP, and AD-related biomarkers. Additionally, our participants were all male and primarily non-Hispanic White, making generalizability to women and racial/ethnic minorities uncertain. This may be relevant given that chronic pain and AD are particularly more prevalent in women than men (62,63); and chronic pain is more undertreated in people identifying as Black and Latino (64).

Regarding our measurement limitations, our measure of chronic pain was not a diagnosis from clinical evaluation and relied on reporting on moderate-to-severe pain in the last 4 weeks across multiple study waves. However, our approach is common in epidemiological literature and shows similar associations as studies using official diagnoses of chronic pain (1,65). Moreover, clinical diagnoses of chronic pain are often based on retrospective recall, while we were able to assess pain longitudinally. Our biomarkers were also limited in that we measured t-tau, which includes a broad array of tau proteins, including those that may be unrelated to AD. Plasma p-tau may be more sensitive and specific to AD-specific processes, and thus more strongly associated with AD-related brain pathology. Furthermore, our measure of inflammation was limited, only measuring hs-CRP, while a more inclusive panel of inflammatory biomarkers may be more informative (eg, interleukin-1B [IL-1B], interleukin-6 [IL-6], and tumor necrosis factor [TNF]) (11,66,67). Though beyond the scope of this study, there are also a host of numerous unmeasured factors, such as lifestyle, diet, and nonopioid medication that might influence both inflammation and neurodegeneration processes. Such analyses may be worthwhile to identify modifiable targets for intervention.

Studies have linked chronic pain to a greater burden of AD risk factors as well as increased risk for ADRD. However, few studies have examined associations with AD-related biomarkers. To our knowledge, only 2 studies have directly examined AD pathology in relation to chronic pain, showing a causal association of chronic pain with neuroinflammation, tauopathy, and hippocampal atrophy (3,5). Our study provides evidence that older men with chronic pain have elevated levels of plasma Aβ42 and Aβ40, likely more a product of systemic inflammation than cerebral amyloidosis. Those with both higher hs-CRP and elevated plasma Aβ also showed the smallest hippocampal volumes. These findings are noteworthy and, while correlational, may shine a light on how systemic inflammation in chronic pain contributes to neurodegeneration in AD-vulnerable regions. Our findings highlight the need for further investigation into the complex interplay of chronic pain, systemic inflammation, and dementia risk.

## Supplementary Material

Supplementary data are available at *The Journals of Gerontology, Series A: Biological Sciences and Medical Sciences* online.

glae206_suppl_Supplementary_Material

## Data Availability

Data are available for research access (https://psychiatry.ucsd.edu/research/programs-centers/vetsa/researchers.html).
