# Supplementary material for: Elevated C-Reactive Protein in Older Men With Chronic Pain: Association With Plasma Amyloid Levels and Hippocampal Volume
Source: J Gerontol A Biol Sci Med Sci. 2024 Aug 22;79(11):glae206. doi: 10.1093/gerona/glae206 (PMC11439493; doi:10.1093/gerona/glae206)
Supplement: glae206_suppl_Supplementary_Material [file glae206_suppl_supplementary_material.docx]

**Supplemental Material**

| Table S1. Mixed model examining the interactive association of chronic pain and hs-CRP level with Aβ42/Aβ40 ratio level. | | | | |
| --- | --- | --- | --- | --- |
|  | **Aβ42/Aβ40** | | | |
| *Predictors* | β | *CI* | *p* |  |
| (Intercept) | -0.03 | -2.06 – 2.00 | .978 |  |
| Chronic Pain | -0.18 | -0.61 – 0.25 | .412 |  |
| hs-CRP | 0.04 | -0.05 – 0.13 | .392 |  |
| CESD Total | -0.01 | -0.02 – -0.00 | **.037** |  |
| Age | 0.00 | -0.03 – 0.03 | .969 |  |
| Physical Morbidities | 0.01 | -0.04 – 0.07 | .602 |  |
| Opioid Use | -0.02 | -0.35 – 0.31 | .908 |  |
| Chronic Pain*hs-CRP | 0.00 | -0.19 – 0.19 | .999 |  |
| **Random Effects** | | | | |
| σ^2^ | 0.72 | | | |
| τ_00_ _CASE_ | 0.26 | | | |
| ICC | 0.26 | | | |
| N _CASE_ | 513 | | | |
| Observations | 792 | | | |
| Marginal R^2^ / Conditional R^2^ | 0.012 / 0.269 | | | |
| Note. hs-CRP = C-reactive protein; ICC = intraclass coefficient, represents the percent of outcome variability explained by people being nested within twin pairs; σ2 = Level 1 error, error across participants; τ00 = Level 2 error, error within twin pairs. Model results did not change when adding *APOE*--ε4 status as a covariate or a moderator (non-significant moderator). | | | | |

| Table S2. Mixed model examining the interactive association of chronic pain and hs-CRP level with t-tau level. | | | |  |  |
| --- | --- | --- | --- | --- | --- |
|  | **t-tau** | | |  |  |
| *Predictors* | β | *CI* | *p* |  |  |
| (Intercept) | 0.48 | -1.21 – 2.17 | .577 |  | |
| Chronic Pain | -0.01 | -0.32 – 0.31 | .971 |  | |
| hs-CRP | -0.00 | -0.07 – 0.07 | .976 |  | |
| CESD Total | 0.00 | -0.01 – 0.01 | .803 |  | |
| Age | -0.01 | -0.03 – 0.02 | .465 |  | |
| Physical Morbidities | 0.04 | -0.00 – 0.08 | .051 |  | |
| Opioid Use | 0.09 | -0.15 – 0.34 | .464 |  | |
| Chronic Pain*hs-CRP | 0.02 | -0.12 – 0.15 | .802 |  | |
| **Random Effects** | | | |  |  |
| σ^2^ | 0.41 | | |  |  |
| τ_00_ _CASE_ | 0.28 | | |  |  |
| ICC | 0.40 | | |  |  |
| N _CASE_ | 554 | | |  |  |
| Observations | 869 | | |  |  |
| Marginal R^2^ / Conditional R^2^ | 0.01 / 0.41 | | |  |  |
| Note. hs-CRP = C-reactive protein; ICC = intraclass coefficient, represents the percent of outcome variability explained by people being nested within twin pairs; t-tau = total tau; σ2 = Level 1 error, error across participants; τ00 = Level 2 error, error within twin pairs. Model results did not change when adding *APOE*--ε4 status as a covariate or a moderator (non-significant moderator). | | | |  |  |

| Table S3. Mixed model examining the interactive association of chronic pain and hs-CRP level with NfL level. | | | | |
| --- | --- | --- | --- | --- |
|  | **NfL** | | | |
| *Predictors* | β | *CI* | *p* |  |
| (Intercept) | -2.81 | -4.34 – -1.28 | **<.001** |  |
| Chronic Pain | 0.00 | -0.28 – 0.28 | .993 |  |
| hs-CRP | 0.05 | -0.01 – 0.11 | .087 |  |
| CESD Total | 0.01 | -0.00 – 0.01 | .126 |  |
| Age | 0.04 | 0.02 – 0.06 | **.001** |  |
| Physical Morbidities | 0.02 | -0.02 – 0.05 | .320 |  |
| Opioid Use | 0.03 | -0.19 – 0.25 | .773 |  |
| Chronic Pain*hs-CRP | 0.06 | -0.07 – 0.18 | .375 |  |
| **Random Effects** | | | | |
| σ^2^ | 0.39 | | | |
| τ_00_ _CASE_ | 0.22 | | | |
| ICC | 0.36 | | | |
| N _CASE_ | 572 | | | |
| Observations | 944 | | | |
| Marginal R^2^ / Conditional R^2^ | 0.03 / 0.38 | | | |
| Note. hs-CRP = C-reactive protein; ICC = intraclass coefficient, represents the percent of outcome variability explained by people being nested within twin pairs; NfL = neurofilament light; σ2 = Level 1 error, error across participants; τ00 = Level 2 error, error within twin pairs. Model results did not change when adding *APOE*--ε4 status as a covariate or a moderator (non-significant moderator). | | | | |

| Table S4. Linear mixed model predicting hippocampal volume in chronic pain from interaction of hs-CRP level and Aβ42 level. | | | |
| --- | --- | --- | --- |
|  | **Hippocampal Volume** | | |
| Predictors | β | CI | *p* |
| (Intercept) | 0.09 | -0.08, 0.25 | .290 |
| Age | -0.03 | -0.13, 0.07 | .512 |
| Physical Morbidities | -0.01 | -0.09, 0.07 | .807 |
| Opioid Use | -0.18 | -0.70. 0.33 | .480 |
| hs-CRP | -0.02 | -0.15, 0.12 | .809 |
| Aβ42 | -0.17 | -0.27, -0.08 | <.001 |
| hs-CRP*Aβ42 | -0.19 | -0.31, -0.07 | .002 |
| Random Effects | |  |  |
| Level 1 Error (σ2) | 0.85 |  |  |
| Level 2 Error (τ00) | 0.05 |  |  |
| ICC | 0.06 |  |  |
| Observations | 331 |  |  |
| Marginal R2 / Conditional R2 | 0.07 / 0.12 | |  |
| Note. Aβ= amyloid-beta; hs-CRP = C-reactive protein; ICC = intraclass coefficient, represents the percent of outcome variability explained by people being nested within twin pairs; σ2 = Level 1 error, error across participants; τ00 = Level 2 error, error within twin pairs. Similar results were found looking at Aβ40 instead. Model results did not change when adding *APOE*--ε4 status as a covariate or a moderator (non-significant moderator). Hippocampal volume was adjusted for estimated intracranial volume. | | | |

| Table S5. Linear mixed model predicting hippocampal volume from interaction of hs-CRP level and Aβ40 level. | | | | |
| --- | --- | --- | --- | --- |
|  | **Hippocampal Volume** | | | |
| *Predictors* | β | *CI* | *p* |  |
| Predictors | -0.14 | -0.47 – 0.20 | .421 |  |
| (Intercept) | -0.04 | -0.17 – 0.09 | .528 |  |
| Age | 0.29 | -0.02 – 0.60 | .063 |  |
| Physical Morbidities | -0.01 | -0.03 – 0.00 | .150 |  |
| Opioid Use | -0.01 | -0.08 – 0.07 | .899 |  |
| hs-CRP | -0.06 | -0.50 – 0.38 | .789 |  |
| Aβ40 | -0.17 | -0.27 – -0.06 | **.002** |  |
| hs-CRP*Aβ40 | -0.22 | -0.35 – -0.08 | **.002** |  |
| **Random Effects** | | | | |
| σ^2^ | 0.86 | | | |
| τ_00_ _CASE_ | 0.03 | | | |
| ICC | 0.04 | | | |
| N _CASE_ | 248 | | | |
| Observations | 332 | | | |
| Marginal R^2^ / Conditional R^2^ | 0.08 / 0.11 | | | |
| Note. Aβ40= amyloid-beta 40; hs-CRP = C-reactive protein; ICC = intraclass coefficient, represents the percent of outcome variability explained by people being nested within twin pairs; σ2 = Level 1 error, error across participants; τ00 = Level 2 error, error within twin pairs. Similar results were found looking at Aβ40 instead. Model results did not change when adding *APOE*--ε4 status as a covariate or a moderator (non-significant moderator). Hippocampal volume was adjusted for estimated intracranial volume. | | | | |

| Table S6. Mixed model examining the interactive association of hs-CRP level and t-tau level with hippocampal volume. | | | | |
| --- | --- | --- | --- | --- |
|  | **Hippocampal Volume** | | | |
| *Predictors* | β | *CI* | *p* |  |
| (Intercept) | -0.13 | -0.47 – 0.22 | .474 |  |
| hs-CRP | -0.10 | -0.21 – 0.02 | .094 |  |
| t-tau | -0.00 | -0.13 – 0.12 | .943 |  |
| White Non-Hispanic | 0.28 | -0.04 – 0.60 | .082 |  |
| CESD Total | -0.01 | -0.03 – 0.00 | .063 |  |
| Physical Morbidities | -0.00 | -0.08 – 0.08 | .943 |  |
| Opioid Use | -0.10 | -0.54 – 0.35 | .665 |  |
| hs-CRP*t-tau | -0.12 | -0.24 – -0.01 | **.048** |  |
| **Random Effects** | | | | |
| σ^2^ | 0.84 | | | |
| τ_00_ _CASE_ | 0.06 | | | |
| ICC | 0.06 | | | |
| N _CASE_ | 241 | | | |
| Observations | 317 | | | |
| Marginal R^2^ / Conditional R^2^ | 0.05 / 0.11 | | | |
| Note. hs-CRP = C-reactive protein; ICC = intraclass coefficient, represents the percent of outcome variability explained by people being nested within twin pairs; t-tau = total tau; σ2 = Level 1 error, error across participants; τ00 = Level 2 error, error within twin pairs. Model results did not change when adding *APOE*--ε4 status as a covariate or a moderator (non-significant moderator). Hippocampal volume was adjusted for estimated intracranial volume. | | | | |

| Table S7. Mixed model examining the interactive association of hs-CRP level and Aβ42/Aβ40 level with hippocampal volume. | | | | |
| --- | --- | --- | --- | --- |
|  | **Hippocampal Volume** | | | |
| *Predictors* | β | *CI* | *p* |  |
| (Intercept) | -0.12 | -0.45 – 0.22 | .494 |  |
| hs-CRP | -0.08 | -0.21 – 0.04 | .195 |  |
| Aβ42/Aβ40 | -.01 | -0.11 – 0.10 | .887 |  |
| White Non-Hispanic | .27 | -.07 – .61 | .126 |  |
| CESD Total | -.02 | -0.04 – 0.00 | .046 |  |
| Physical Morbidities | -0.00 | -0.09 – 0.08 | .927 |  |
| Opioid Use | -.14 | -0.62 – 0.34 | .559 |  |
| hs-CRP*Aβ42/Aβ40 | .02 | -0.08 – 0.13 | .671 |  |
| **Random Effects** | | | | |
| σ^2^ | 0.99 | | | |
| τ_00_ _CASE_ | 0.03 | | | |
| ICC | 0.03 | | | |
| N _CASE_ | 247 | | | |
| Observations | 331 | | | |
| Marginal R^2^ / Conditional R^2^ | 0.03 / 0.03 | | | |
| Note. Aβ42/Aβ40 = ratio of amyloid-beta 42 and amyloid-beta 40; hs-CRP = C-reactive protein; ICC = intraclass coefficient, represents the percent of outcome variability explained by people being nested within twin pairs; σ2 = Level 1 error, error across participants; τ00 = Level 2 error, error within twin pairs. Model results did not change when adding *APOE*--ε4 status as a covariate or a moderator (non-significant moderator). Hippocampal volume was adjusted for estimated intracranial volume. | | | | |

| Table S8. Mixed model examining the interactive association of hs-CRP level and NfL level with hippocampal volume. | | | | |
| --- | --- | --- | --- | --- |
|  | **Hippocampal Volume** | | | |
| *Predictors* | β | *CI* | *p* |  |
| (Intercept) | -0.09 | -0.42 – 0.25 | .608 |  |
| hs-CRP | -0.05 | -0.17 – 0.07 | .419 |  |
| NfL | -0.14 | -0.27 – -0.01 | **.039** |  |
| White Non-Hispanic | 0.30 | -0.02 – 0.61 | .064 |  |
| CESD Total | -0.02 | -0.03 – -0.00 | **.032** |  |
| Physical Morbidities | -0.02 | -0.10 – 0.07 | .694 |  |
| Opioid Use | -0.30 | -0.74 – 0.14 | .181 |  |
| hs-CRP*NfL | -0.08 | -0.23 – 0.08 | .350 |  |
| **Random Effects** | | | | |
| σ^2^ | 0.94 | | | |
| τ_00_ _CASE_ | 0.00 | | | |
| ICC | 0.00 | | | |
| N _CASE_ | 246 | | | |
| Observations | 348 | | | |
| Marginal R^2^ / Conditional R^2^ | 0.06 / 0.06 | | | |
| Note. hs-CRP = C-reactive protein; ICC = intraclass coefficient, represents the percent of outcome variability explained by people being nested within twin pairs; NfL = neurofilament light; σ2 = Level 1 error, error across participants; τ00 = Level 2 error, error within twin pairs. Model results did not change when adding *APOE*--ε4 status as a covariate or a moderator (non-significant moderator). Hippocampal volume is adjusted for estimated intracranial volume. | | | | |

| Table S9. | | | | | | |
| --- | --- | --- | --- | --- | --- | --- |
|  |  | Aβ42/Aβ40 | Aβ42 | Aβ40 | t-tau | NFL |
| Chronic Pain*hsCRP* | | *p* | *p* | *p* | *p* | *p* |
| Pain Interference | | .902 | .083 | .309 | .460 | .209 |
| Depressive Symptoms | | .915 | .794 | .473 | .891 | .411 |
| MCI Status | | .431 | .866 | .971 | .050 | .114 |
| *APOE*-ε4 status | | .072 | .844 | .274 | .719 | .525 |
| Note. Aβ42= amyloid-beta 42; Aβ40= amyloid-beta 40; hs-CRP = high sensitivity C-reactive protein; MCI = mild cognitive impairment; NfL = neurofilament light; t-tau = total tau. | | | | | | |

| Table S10. | | | | |
| --- | --- | --- | --- | --- |
|  |  |  | Hippocampal Volume | |
|  | | | *p* |  |
| Chronic Pain*hsCRP*Aβ42/Aβ40* | | |  |  |
| Functional Interference | | | .208 |  |
| Depressive Symptoms | | | .596 |  |
| *APOE*-ε4 status | | | .209 |  |
| MCI Status | |  | .899 |  |
|  |  |  |  |  |
| Chronic Pain*hsCRP*Aβ42* | | |  |  |
| Functional Interference | | | .612 |  |
| Depressive Symptoms | | | .162 |  |
| *APOE*-ε4 status | | | .122 |  |
| MCI Status | |  | .629 |  |
|  |  |  |  |  |
| Chronic Pain*hsCRP*Aβ40* | | |  |  |
| Functional Interference | | | .699 |  |
| Depressive Symptoms | | | .271 |  |
| *APOE*-ε4 status | | | .221 |  |
| MCI Status | |  | .766 |  |
|  | |  |  |  |
|  |  |  |  |  |
| Chronic Pain*hsCRP*-t-tau* | | |  |  |
| Functional Interference | | | .365 |  |
| Depressive Symptoms | | | .302 |  |
| *APOE*-ε4 status | | | .450 |  |
| MCI Status | |  | .610 |  |
|  | |  |  |  |
|  |  |  |  |  |
| Chronic Pain*hsCRP*NFL* | | |  |  |
| Functional Interference | | | .794 |  |
| Depressive Symptoms | | | .939 |  |
| *APOE*-ε4 status | | | .130 |  |
| MCI Status | |  | .215 |  |
| Note. Aβ42= amyloid-beta 42; Aβ40= amyloid-beta 40; hs-CRP = high sensitivity C-reactive protein; MCI = mild cognitive impairment; NfL = neurofilament light; t-tau = total tau. Hippocampal volume was adjusted for estimated intracranial volume. | | | |  |
|  |  |  |  |  |


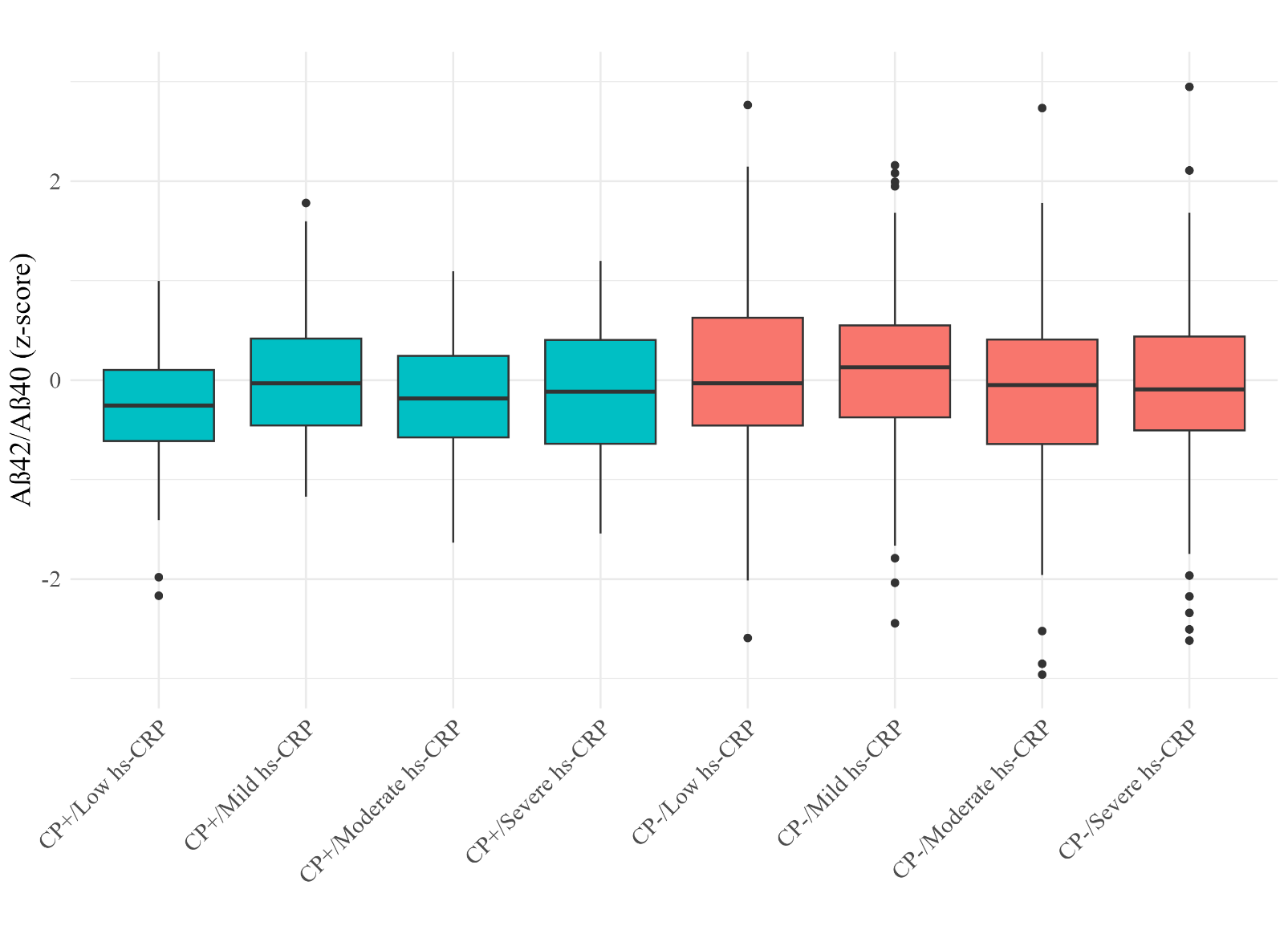


Figure S1. Differences in Aβ42/Aβ40 level by severity of hs-CRP level in men with chronic pain (left panel) and without chronic pain (right panel) (*n* = 792). Note. Aβ42/Aβ40 = ratio of amyloid-beta 42 and amyloid beta 40; hs-CRP = high-sensitivity serum C-Reactive Protein. Aβ42/Aβ40 values were z-scored for ease of interpretability. hs-CRP levels represent tertiles of low (0 to .40 mg/dL), mild (.40 to 2.60 mg/dL), moderate (2.61 to 4.70 mg/dL) and high values (4.71 to 19.00* mg/dL, *Outliers winsorized at 3 *SD* or 19.00). Lower levels of Aβ42/Aβ40 are indicative of greater cerebral amyloid pathology as Aβ42 is deposited in the brain.


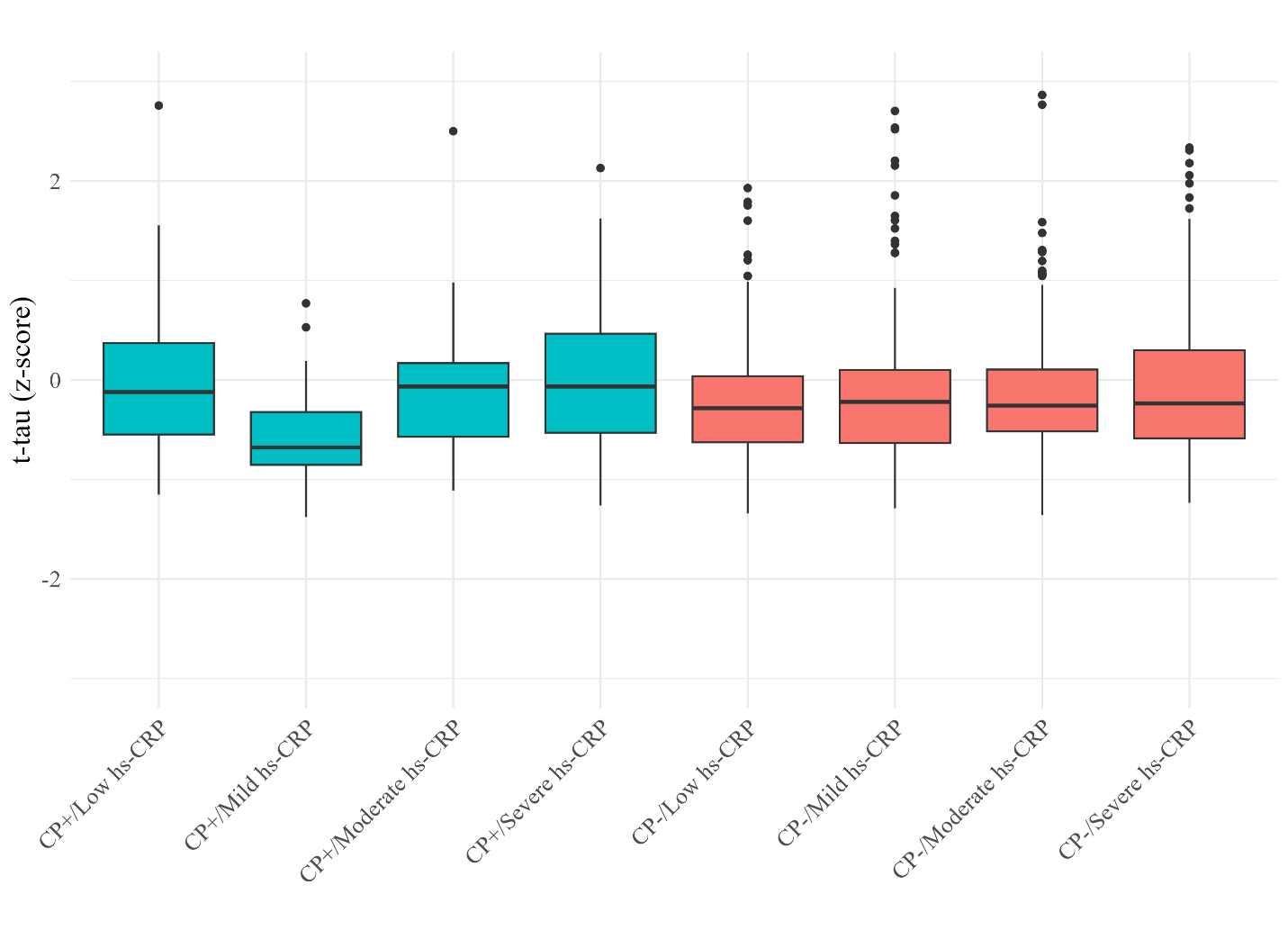


Figure S2. Differences in t-tau level by severity of hs-CRP level in men with chronic pain (left panel) and without chronic pain (right panel) (*n* = 869). Note. t-tau = total tau. t-tau values were z-scored for ease of interpretability. hs-CRP levels represent tertiles of low (0 to .40 mg/dL), mild (.40 to 2.60 mg/dL), moderate (2.61 to 4.70 mg/dL) and high values (4.71 to 19.00* mg/dL, *Outliers winsorized at 3 *SD* or 19.00).


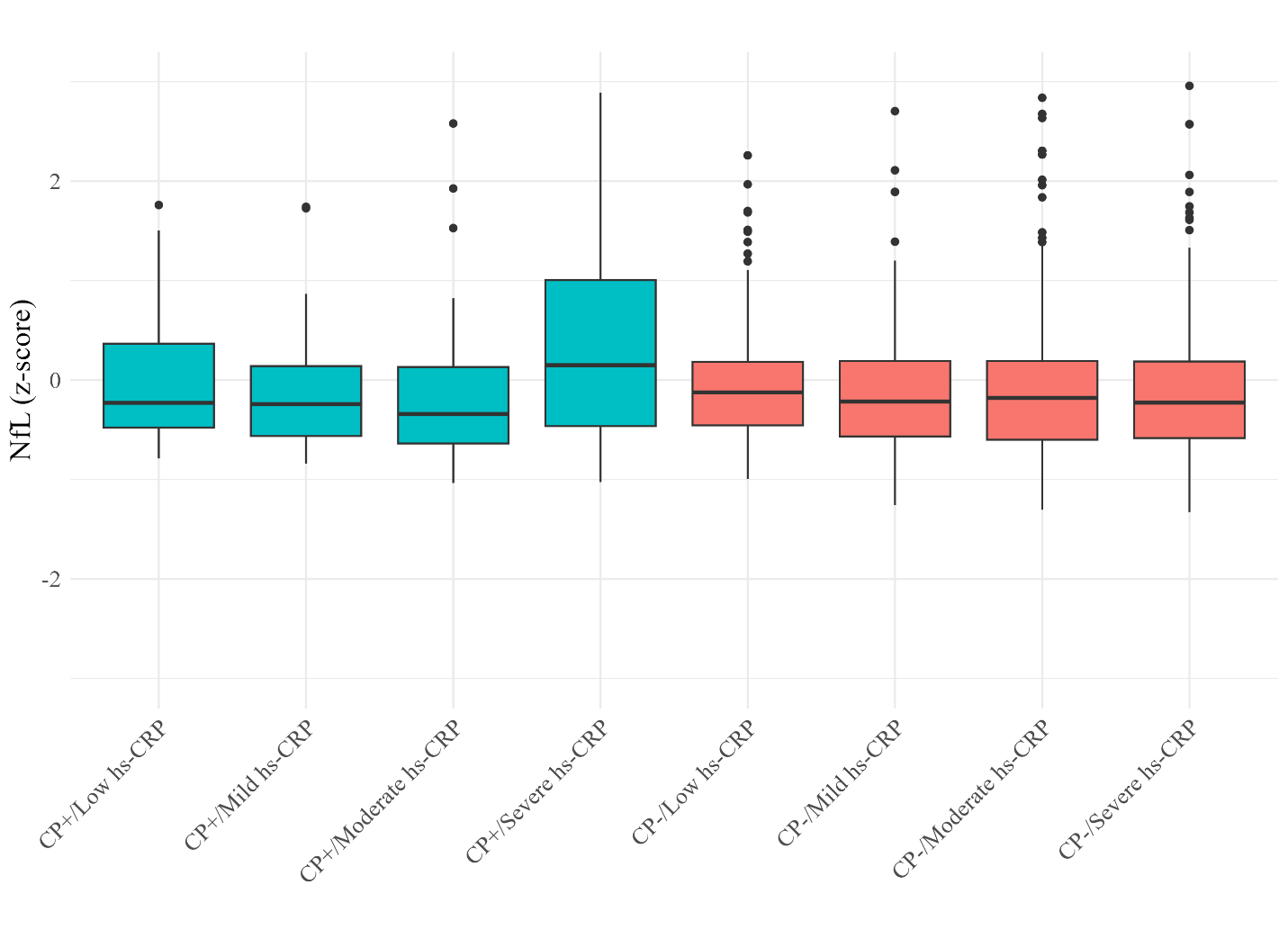
Figure S3. Differences in NfL level by severity of hs-CRP level in men with chronic pain (left panel) and without chronic pain (right panel) (*n* = 944). NfL values were z-scored for ease of interpretability. hs-CRP levels represent tertiles of low (0 to .40 mg/dL), mild (.40 to 2.60 mg/dL), moderate (2.61 to 4.70 mg/dL) and high values (4.71 to 19.00* mg/dL; *Outliers winsorized at 3 *SD* or 19.00).


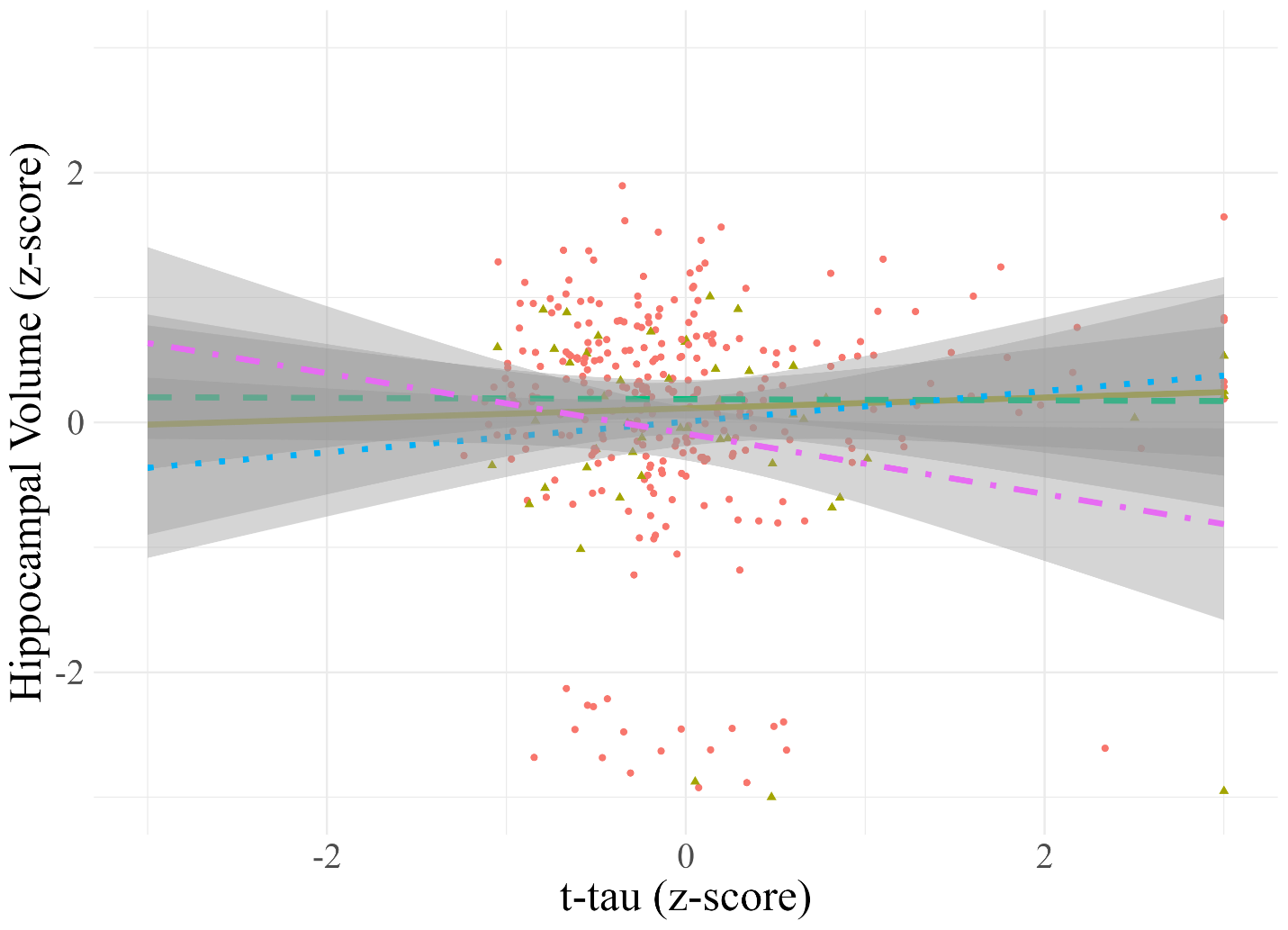


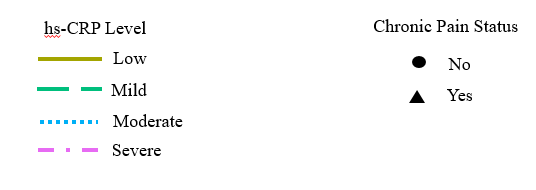


Figure S4. Association of t-tau and hippocampal volume by different levels of hs-CRP (*n* = 317). Note. hs-CRP = high sensitivity serum C-reactive protein; t-tau = total tau. hs-CRP levels represent tertiles of low (0 to .40 mg/dL), mild (.40 to 2.60 mg/dL), moderate (2.61 to 4.70 mg/dL) and high values (4.71 to 19.00* mg/dL, *upper values winsorized at 3 *SD* or 19.00). hs-CRP, t-tau, and hippocampal volume values were z-scored for ease of interpretability.


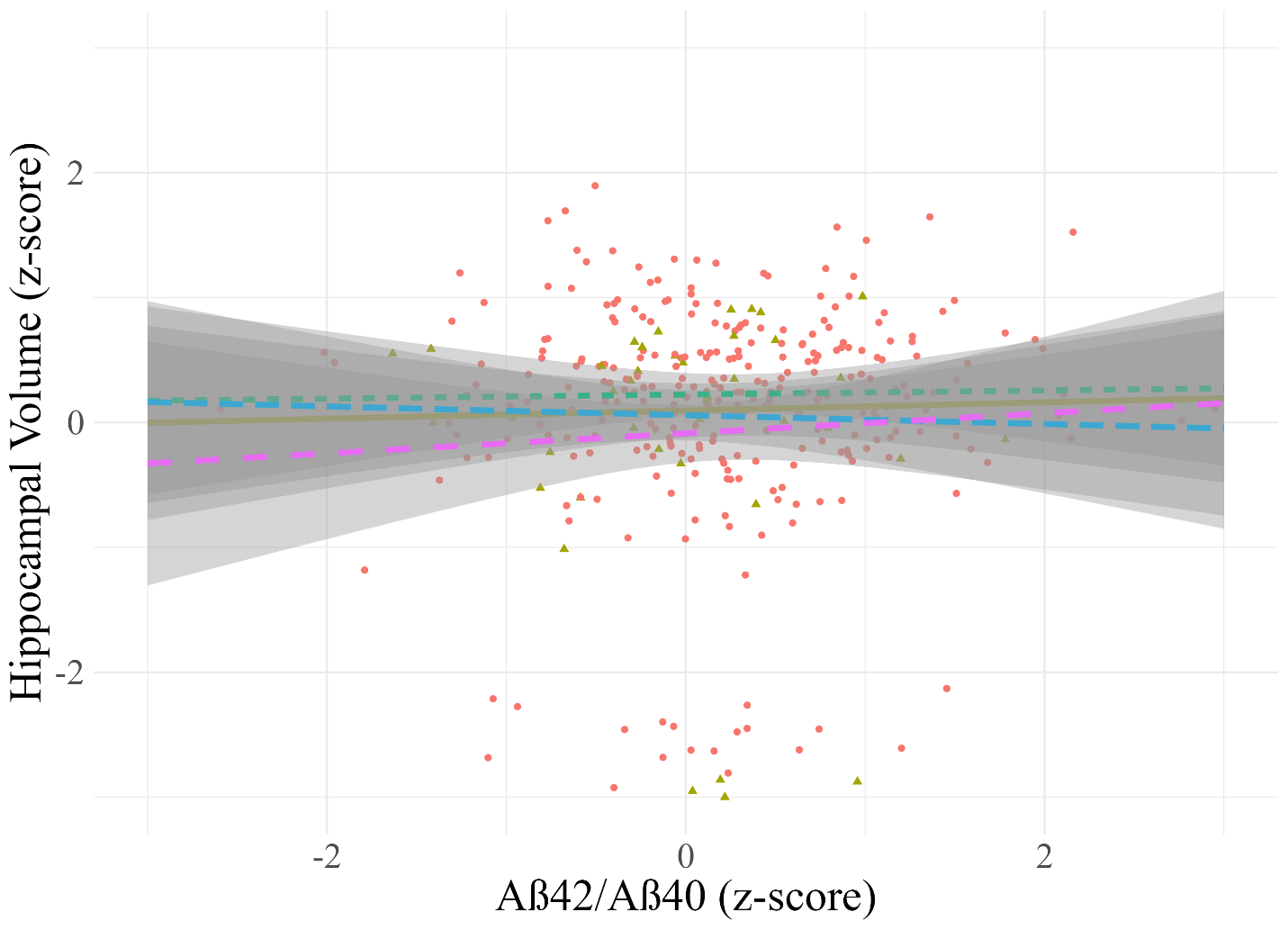


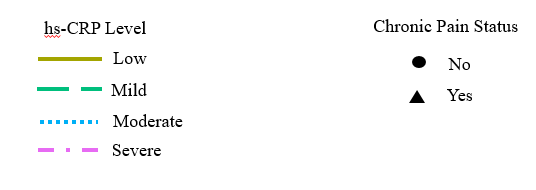


Figure S5. Association of Aβ42/Aβ40 with hippocampal volume by different levels of hs-CRP (*n* = 331). Note. Aβ42 = amyloid-beta 42; Aβ40 = amyloid-beta 40; hs-CRP = high sensitivity serum C-reactive protein. hs-CRP levels represent tertiles of low (0 to .40 mg/dL), mild (.40 to 2.60 mg/dL), moderate (2.61 to 4.70 mg/dL) and high values (4.71 to 19.00* mg/dL, *upper values winsorized at 3 *SD* or 19.00). hs-CRP, Aβ42/Aβ40, hippocampal volume values were z-scored for ease of interpretability.


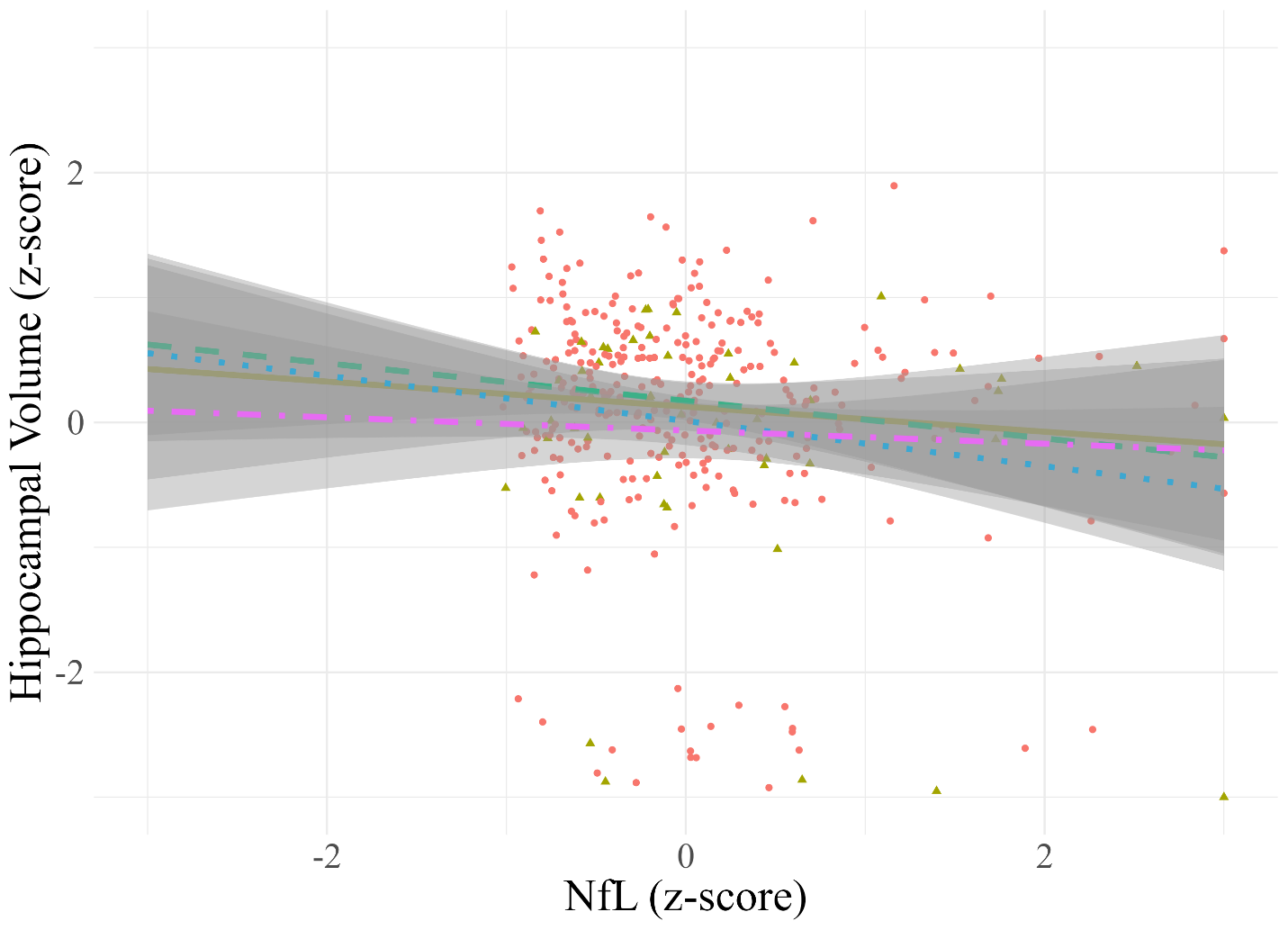


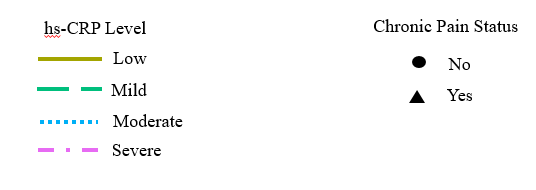


Figure S6. Association of NfL and hippocampal volume by different levels of hs-CRP (*n* = 371). Note. hs-CRP = high sensitivity serum C-reactive protein; NfL = neurofilament light. hs-CRP levels represent tertiles of low (0 to .40 mg/dL), mild (.40 to 2.60 mg/dL), moderate (2.61 to 4.70 mg/dL) and high values (4.71 to 19.00* mg/dL, *upper values winsorized at 3 *SD* or 19.00). hs-CRP, NfL, and hippocampal volume values were z-scored for ease of interpretability.
